# Enhancing Executive Functions Among Dutch Elementary School Children Using the Train Your Mind Program: Protocol for a Cluster Randomized Trial

**DOI:** 10.2196/resprot.7908

**Published:** 2018-06-07

**Authors:** Joachim Bervoets, Lisa M Jonkman, Sandra Mulkens, Hein de Vries, Gerjo Kok

**Affiliations:** ^1^ Department of Work & Social Psychology Maastricht University Maastricht Netherlands; ^2^ Department of Cognitive Neuroscience Maastricht University Maastricht Netherlands; ^3^ Department of Clinical Psychological Science Maastricht University Maastricht Netherlands; ^4^ Department of Health Promotion and Education Maastricht University Maastricht Netherlands

**Keywords:** executive function, children, socioemotional development, cognitive development, academic performance, physical activity, healthy eating, eHealth

## Abstract

**Background:**

Executive functions are higher cognitive control functions, which are essential to physical and psychological well-being, academic performance, and healthy social relationships. Executive functions can be trained, albeit without broad transfer, to this date. Broad transfer entails the translation of improved cognitive functions to daily life (behaviors). The intervention Train your Mind was designed to train executive functions among elementary school children aged 9 to 11 years, and obtain broad transfer in terms of enhanced physical activity, healthy eating, and socioemotional regulation.

**Objective:**

This paper aims to describe the cluster randomized trial to test the effectiveness of the Train your Mind intervention.

**Methods:**

Train your Mind was integrated into the existing school curriculum for 8 months (25 weeks excluding holidays). The effectiveness of the intervention was tested in a cluster randomized trial comprising 13 schools, 34 groups (school classes), and 800 children, using a battery of 6 computer tasks at pre- and postmeasurement. Each of the 3 core executive functions was measured by 2 tasks (Flanker and Go/No-Go; N-Back and Running Span; Attention Switching Task and Dots/Triangles). Moreover, we administered questionnaires that measure emotion-regulation, cognitive errors, physical activity, dietary habits, and the psycho-social determinants of diet and physical activity. Body mass index was also measured. Multilevel analyses will account for clustering at the school and group levels, and randomization took place at the school level.

**Results:**

Results are currently being analyzed.

**Conclusions:**

The main purpose of this study is to test Train your Mind’s effectiveness in enhancing executive functions. Second, we investigate whether increased executive functions lead to improved physical activity and healthy eating. If found effective, executive function training could easily be integrated into school curricula everywhere, and as such, boost health, academic performance, and emotion-regulation of elementary school children, in a cost-effective manner.

**Trial Registration:**

Netherlands Trial Register NTR5804; http://www.trialregister.nl/trialreg/admin/rctview.asp?TC=5804 (Archived by WebCite at http://www.webcitation.org/6z9twosJ8)

**Registered Report Identifier:**

RR1-10.2196/7908

## Introduction

### Background

Executive functions (EFs) are higher mental control functions, consisting of impulse control, working memory and cognitive flexibility [1,2]. EFs are vital to a myriad of areas in life, including physical [3,4] and psychological well-being [5], academic performance [6], and healthy social relationships [7]. EFs can be trained [8,9]. However, such strengthened cognitive functions have, as of yet, not been accompanied by meaningful, behavioral improvements in daily life—otherwise known as broad transfer [10,11]. Recent literature suggests (combining) multiple approaches to train EFs and subsequently attain broad transfer [2,10]. Train your Mind (TyM) follows these recommendations, and as such, applies 3 main modules to train EFs: (1) focused physical activity, (2) cognitive games, and (3) socioemotional development. A fourth module, (4) tailored feedback (eHealth), aims to directly change the behaviors physical activity and healthy eating. These modules are presented in great depth in a separate paper, including the development and implementation (J Bervoets et al, unpublished data, 2018). An extensive background on what EFs are, how they can be trained, and why they are so pivotal to a healthy and successful life is described in the same separate paper (J Bervoets et al, unpublished data, 2018). This paper focuses on the study design and outcome measures.

### Aims and Hypotheses

This study’s main objective is to enhance EFs, among 9- to 11-year-old elementary school children, through a multimodule intervention combining physical, cognitive, and socioemotional approaches. Longitudinal EF studies have revealed a substantial amount of positive correlates, including physical and mental well-being, level of education, job income, marital harmony, substance use, unsafe sex, risky driving, eating, and social relations [2,4,5]. This lead us to expect improved EFs to translate into healthier behavior in daily life (especially considering our multidimensional, integrated intervention), more specifically, in our target population. As a result of stronger EFs, we expected the children to be physically more active and eat more healthily. Such daily life improvements as a result of strengthened EFs would constitute the much-desired broad transfer. This broad transfer is hypothesized to be mediated by EFs. To bridge the seemingly distant cognitive control functions with physical activity and eating, it may help to consider self-regulation, concentration, and attention. It is easier to envision how the latter are improved as a result of stronger EFs (eg, inhibitory control can shut out distracting stimuli, keeping the mental working space clear, but also maintaining optimal levels or emotional and cognitive arousal, by self-regulation). In sum, the model we are working with assumes a 3-level cascade effect: (1) increased EFs will lead to (2) improved self-control, emotion-regulation, and attention, which in turn will facilitate (3) healthy eating and physical activity.

#### Main Hypothesis

The main hypothesis is as follows:

- EFs are enhanced through a multi-dimensional intervention (TyM).

#### Secondary Hypotheses

The secondary hypotheses are as follows:

TyM effects transfer to improved:

- Emotional-regulation
- Concentration/attention

TyM effects translate to other aspects of daily life, specifically:

- More physical activity
- Healthier dietary habits
- Reduced social/emotional/behavioral problems
- Better school performance
- These broad transfer effects are mediated by stronger EFs

## Methods

### Trial Design

The effectiveness of TyM was investigated in a cluster randomized trial comprising 13 elementary schools, with a total of 34 groups and 800 children. Schools in the control condition continued their regular school curriculum, and would receive the program, or any components thereof, after the third and final measurement (follow-up) had been completed. In this sense, it was a passive control group (though the same amount of time will be spent on similar activities, gym sessions, etc, minus the explicit EF training). All groups of the Dutch sixth and seventh grade of schools in the experimental condition followed the entire TyM intervention for 8 months (25 weeks excluding holidays). This means it is nearly impossible to determine effectiveness per component—our study was set up primarily to answer the question: Can EFs be enhanced through a multi-dimensional training program? If satisfactory results are obtained, TyM can be disentangled in the future to further scrutinize each component separately. Children were to be measured at 3 different time points: preintervention, postintervention, and at follow-up 6 months later. However, the follow-up could not be organized due to financial constraints.

### Participants and Procedure

The promising potential that lies in training EFs (academic performance, healthy behaviors, better social relations) was presented to education foundations (Movare and Innovo) in South-Limburg, The Netherlands. Interested school directors and teachers further inquired about the willingness of other teachers to participate. A total of 13 schools, 34 groups, representing 800 pupils, decided to participate in TyM. Every sixth or seventh grade pupil (aged 9-11 years) of a participating school automatically took part in the intervention activities, as they were integrated in the existing school curriculum. Participation in measurements, on the other hand, could be refused by parents. Every child of the relevant school classes was welcome to join; we assumed normally distributed groups in terms of attention problems and fluid intelligence.

### Ethics and Consent to Participate

Consent was obtained from the schools. Both parents and children were informed about the intervention and measurements, and both parents (written) and children (verbal) were asked to provide consent. Parents and students can withdraw from participation at any time. This intervention, along with the study methods and consent procedure, were approved by the Ethical Review Committee of the Faculty of Psychology and Neuroscience, Maastricht University, the Netherlands (dd. 13-08-2015, ERCPN-06-06-2015).

### Availability of Data and Materials

Data are not yet available. Materials can freely be requested from j.bervoets@maastrichtuniversity.nl.

### Randomization

Schools, matched by number of pupils in the participating grades, and from similar areas, were randomly assigned to either an experimental or a control condition, by means of a coin toss (simple randomization) performed by the first author of this paper. Before this process, however, 3 schools had to be assigned a condition because they would not participate otherwise. One school could only participate as a control school because they had large infrastructure works scheduled for the school year and did not want to overly burden the teachers in that hectic period. Two schools were so interested in training EFs they would only agree to participate as experimental schools, as they wanted to train EFs regardless (they could not guarantee respecting the terms of being a control school). We decided to include all the 3 schools anyway, because in any case, it would offer us valuable insights in how our novel and ambitious intervention would work in the field, especially at highly motivated schools. Because it had not yet been (sufficiently) proven in previous studies that EFs can be trained, we decided to maximize our chances of finding an answer to the most fundamental research question of our project: can EFs be trained? (by TyM). Furthermore, all schools were highly motivated to participate. This flaw in the randomization process will be taken into account during analysis.

### Intervention

TyM combines (1) focused physical activity, (2) cognitive games, and (3) socioemotional development in an attempt to enhance EFs. Physical activity and healthy eating are directly targeted in a fourth module (4) tailored feedback (eHealth). To this date, it has proven challenging to sustainably train EFs, and even more so to achieve broad transfer [10]. Leading experts in the field suggest considering novel angles such as the mind-body connection and socioemotional balance [2,10,12-15]. The development, implementation, and content of these modules are described in great detail in a separate paper (J Bervoets et al, unpublished data, 2018). A brief description of the intervention is presented in the following paragraphs.

Promising results with regard to training EFs were found by a yoga [15] and a taekwondo intervention [13,14]. For TyM, core elements of kung fu were comprised into the focused physical activity component, as the fundamental idea of kung fu is self-control (achieved through rigorous training aimed at intrapersonal progress—it is by nature not competitive). After initial workshops (for teachers) and introductory lessons (from our kung fu expert to the participating class groups), teachers lead the kung fu sessions themselves, during physical education hours at school (1 hour/week), supported by a teaching manual.

Despite an apparent lack of groundbreaking results for cognitive training within the field of EF training [2,10,16], we reasoned that this angle still merited a place in our multidimensional intervention. The idea was that effects of singular components could multiply synergistically in an integrated whole. Moreover, approaches could be combined in applications, such as cognitive and socioemotional training while playing board games (handle losing, cheating). The TyM collective cognitive games included (1 hour/week): SET, charades, taboo, and memory. Theoretically, the most appropriate games for EF training are challenging and engage multiple EF subcomponents simultaneously [2,8] (eg, SET).

Furthermore, to ensure an incremental challenge at the individual level, individual Web-based games were presented (Cambridge Brain Sciences), including adapted Raven’s progressive matrices, Stroop, paired-associate learning, Hampshire tree task, and spatial working memory/planning.

An effective guide to address socioemotional development while training EFs was found in MindUp [17], in which children learn to recognize and handle emotions in a more functional way [18,19]. The core technique of mindful breathing aids children in finding and maintaining their composure. Pupils learn more about the relationships between thoughts, emotions, and behavior. We adapted the existing MindUp program to our own Dutch target population. All schools already had some kind of socioemotional development program (and allotted weekly time slot), which was replaced by our unified version of MindUp (30 minutes/week for the duration of the trial (25 weeks, excluding holidays), including what we believed were essential components (such as mindful breathing). A teaching manual with additional background information, suggested lesson structures, and hints supported the teachers for these sessions.

The latter 3 modules target EF training, whereas our fourth TyM module aims to change behavior directly; personalized feedback/advice regarding physical activity and healthy eating is generated based on psycho-social determinants that are assessed in the Web-based eHealth module. The feedback is construed through behavior change models such as Reasoned Action Approach [20] and Theory of Planned Behavior [21].

Control groups continued their regular curricula.

### Primary Outcomes

At the core of our intervention and study lied EFs. These EFs were measured using a computerized task battery consisting of 6 tasks (2 per EF subcomponent), consistent with most recent suggestions from the field [2,22,23]. Careful attention was paid to the fact that testing and training tasks must be adequately different, so as to avoid practice effects (ie, merely measuring children’s improvement on 1 [near-] similar task that they have repeated/practiced over and over).

 In terms of the measurements themselves, we hoped that any practice effects would be minimized by the long periods of time in between pre, post, and follow-up measurements (7 and 6 months, respectively), by the fact that there was a control group and, most importantly, by the fact that the measurement tasks were sufficiently different from the (Web-based) cognitive training tasks. Both original tasks and adapted versions of existing tasks were included in this task battery. The entire battery was pilot-tested in a small study investigating the effectiveness of a 6-session kung fu intervention designed to enhance EFs. As a result, the task battery was fine-tuned and any final glitches were ironed out; it takes about 1 hour and 15 min to complete it the first time. In the section below, we provide an overview and descriptions of the tasks per EF subcomponent.

### Secondary Outcomes

As the development of EFs is boosted, we hypothesize various daily life behaviors to benefit from this as well (see secondary hypotheses for an overview of the aspects this study focused on). These outcomes constitute the aforementioned broad transfer and practical relevance of increased EFs; other than stronger EFs, what are the potential benefits for the individual? The case for physical activity and healthy eating is peculiar, as they can not only be affected by increased EFs but also by the tailored feedback eHealth module, which specifically targets these 2 behaviors. 

The eHealth module operates a behavior change model that is almost entirely driven by determinants (of the targeted behaviors), assisted by additional behavior change techniques such as coping plans. These determinants are measured by the program, during the intervention, as personalized feedback is offered to the individual, in response to these determinants and how they evolve over time. However, the very same determinants are also included in the large pre and post-intervention measurements, to allow for comparison with the control group. Measuring these determinants allows us to investigate what changes in determinants drove the change in behavior—useful knowledge in light of similar future behavior change interventions. An overview of the outcome measures is presented in Textbox 1.

#### Executive Functions Battery

- Inhibitory/interference control: (1) Flanker and (2) Go/No-Go
- Working memory/updating: (3) N-Back and (4) Running Span
- Cognitive flexibility/switching: (5) Attention Switching Task (AST) and (6) Dots/Triangles

Stimulus acquisition and presentation in all 6 tasks are controlled by Presentation software. Response button boxes (Cedrus RB-844), connected to 30 identical Hewlett-Packard laptops, registered responses very accurately.

##### Flanker Task

The current task battery makes use of an adapted version of the original Eriksen Flanker task [24] to measure children’s response interference control abilities. In this adapted version, letters are used instead of arrows to avoid the well-documented developmental ceiling effects associated with the traditional arrow flanker task, which is found at around 10 years of age in normally developing children [25].

 An array of 3 letters is presented at the center of the screen. The middle letter is the target letter and the other 2 are flankers. Participants are instructed to press the left button on the Cedrus RB-844 response button box when the target letter is a B or H, and the right button if the target is letter F or T—while ignoring the flanking stimuli (two B’s, H’s, F’s, or T’s).

**Textbox 1. Overview of the outcome measures.**

**Primary Outcomes**

- Executive functions: Computer task battery
  - Working memory: N-Back; Running Span
  - Impulse control: Flanker; Go/No-Go
  - Cognitive flexibility: Attention switching task; Dots/triangles

**Secondary Outcomes**

- Emotion-regulation, attention, healthy behaviors: Survey
  - Emotion-regulation: FEEL-KJ (Fragebogen zur Ehrebung der Emotionsregulation bei Kindern und Jugendlichen; Questionnaire for the Assessment of Emotion Regulation among Children and Adolescents)
  - Mental health—social/emotional/behavioral functioning: Strengths and Difficulties Questionnaire
  - Attention-Related Cognitive Errors (Scale; ARCES)
  - Physical Activity Questionnaire (PAQ-C)
  - Dietary habits
  - Determinants of dietary habits and physical activity
  - Demographics
- Additional data from provincial database: socioeconomic status and academic performance (CITO; Centraal Instituur voor Toetsontwikkeling; Central Institute for the Development of Tests; a standardized test of academic performance, widely used in the Netherlands)

There are 3 stimulus conditions:

1. Stimulus-response congruent (C) condition: no response conflict (eg, B B B)
2. Stimulus incongruent (SI) condition: target and flankers are different letters but both are mapped to a response of the same hand so that there is stimulus conflict but no response conflict (B H B; H B H; F T F; T F T)
3. Stimulus and response incongruent (SRI) condition in which the flankers and target differ in both the physical appearance (stimulus conflict) and the required response hand (response conflict; eg, F B F; H T H).

A total number of 144 trials are presented in 3 blocks of 48 trials. Each of the 3 conditions is randomly presented with equal probability within task blocks. Each trial starts with the presentation of 2 flanking stimuli for 200 ms (to establish flanker priming effects), after which the middle target letter appears and the entire array of 3 letters stays on the screen for 700 ms, followed by a fixed interstimulus interval (ISI) of 500 ms, during which a fixation cross is presented. The maximum response window is 1400 ms. The experimental task (no feedback) is preceded by a practice block of 18 trials in which the feedback: “correct,” “wrong,” or “faster” is given. To measure the executive function *response interference control* the accuracy and reaction time of participants in the C and SRI conditions will be compared.

##### Go/No-Go

In the current Go/No-Go task, the participants are presented with a random series of letters drawn from the alphabet and appearing one by one in the center of the computer screen. The participants’ task is to respond to every letter by pressing the right button on a Cedrus RB-844 response box (Go stimuli), but to refrain from responding when they see the letter X (the No-Go stimulus). Each trial lasts 1200 ms, with a stimulus presentation rate of 500 ms and a fixed ISI of 700 ms. A total of 200 trials are presented in 2 blocks of 100, with a pause in between. Within each task, the probability that a No-Go stimulus will appear is 10.0% (main task: twice 10/100). The experimental task (no feedback) is preceded by a text instruction screen and 15 practice trials (feedback is only given when a participant incorrectly responds to the No-Go stimulus). The Go/No-Go task has been widely used to study the development of response inhibition [26].

##### N-Back

This version of the N-back task has been used to investigate the development of nonspatial working memory capacity among children and adolescents [27]. Semirandom sequences of letters (A, B, F, G, H, K, L, S, T, W, X, Z) appear one at a time in the center of the screen. Letters (height: 1 cm, width: 0.5 cm) are black and presented between 2 black vertical bars (height: 1.5 cm) on a gray background. The current N-back task consists of 1- and 2-back conditions. Participants are instructed to respond by pressing a Cedrus button box RB-844, with their right index finger, whenever they detect a target event. In 1- and 2-back conditions a target is, respectively, defined as a letter that is identical to the letter presented 1 (eg, T-T), or 2 trials back in the sequence (eg, A-B-A). The experimental task consists of a total of 200 trials, presented in 4 blocks of 50 stimuli, 2 blocks per condition. The order of the block presentation is: 1-back – 2-back – 1-back – 2-back. All blocks have an identical target frequency of 36% (18/50). Each trial lasts 2000 milliseconds (ms) with a stimulus duration of 500 ms and a fixed ISI of 1500 ms. The experimental tasks are preceded by several instruction screens with pictures explaining the specific task requirements, directly followed by 20 practice trials (target event frequency 40%, 8/20) during which feedback is given (“wrong” for false alarms and “missed” when a target-event is not detected). Due to the complexity of the 2-back blocks, a second practice session of 20 trials is automatically run when the success rate in the first practice block falls below 70% (14/20). No feedback is given during the experimental/real tasks.

##### Running Span Task

In this task, a series of numbers varying in length are presented one by one at the center of the computer screen. Each number series is preceded by “The next rows of numbers will follow. If you are ready, press ENTER,” after which the series starts. Within a series, each digit is presented for 1 s with an interval of 500 ms between them. Following the presentation of the entire sequence, participants are presented with a screen showing the presented sequence with the last 3 numbers displayed as 3 question marks. The participants have to recall these 3 missing numbers from working memory and type them in the spaces indicated by the question marks by using the number buttons on the laptop keyboard and ENTER when finished. For example: if series 4, 2, 5, 8, 3, and 9 is presented, the participant sees “4 2 5 ? ? ?” on the screen and has to enter 8, 3, 9 in the spaces represented by the question marks.

In the present task, 12 number series are presented that differ in length; there are 4 series each of 5, 6, and 7 digits that are presented randomly so that participants cannot predict series length. In each case, the last 3 digits of a series have to be recalled. The experimental part of the task—during which no feedback is given—is preceded by instruction screens explaining the task followed by 2 practice trials with feedback. The dependent measures in this task are the mean percentage of correctly completed trials per series length and the average time at which the enter button is pressed once the missing numbers are entered. A similar version of this running span, the digit memory task, has been used before to measure updating in children [28].

##### Attention Switching Task (AST)

The present task is an adapted version of the task previously used in 2 developmental studies by Cepeda et al [29,30]. The task consists of 3 blocks: 2 nonswitch blocks (2 x 32 trials) precede 1 switch block (64 trials). On each trial, 1 of the 4 possible stimulus displays is presented (1, 111, 3, or 333). In the first block, the instruction “which number?” precedes the stimulus 1 or 111 and is correctly responded to by pressing the left button on the Cedrus RB-844 button box (and right button for 3 or 333). In the second block (“how many numbers?”), the left response button represents the single digits (1 and 3), whereas the right button is associated with a string of 3 digits (111 or 333), irrespective of the identity of the digits. In the third block (the switch block), cue instructions (“which number?” or “how many numbers?”) alternate randomly, meaning that participants have to switch between instructions on about half of the trials. Every trial starts with the presentation of the cue, followed by the stimulus after 200 ms. Both remain on the screen until a response is given. Each experimental block commences with instruction screens explaining the task and allowing the participants to practice each stimulus-response configuration. Feedback is given during the entire task (“wrong” or “faster,” no feedback for correct responses).

##### Dots/Triangles

**Figure 1:**
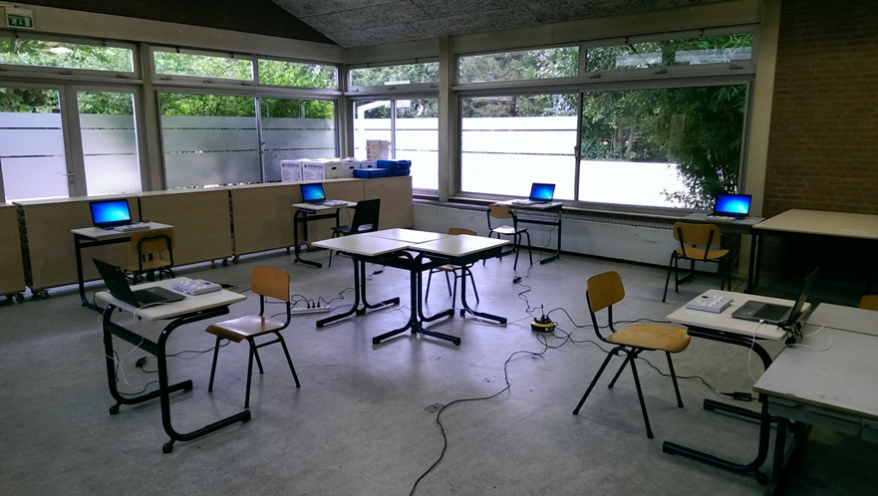
Example of an executive function (EF) testing station setup at an elementary school.

This task is adapted from the original [22] in several ways: (i) Diagram instruction screens have been inserted, (ii) feedback is given after every trial during the practice period (for 1000 ms) rather than after the entire block, (iii) no feedback is given during the experimental blocks, (iv) a minimum for the difference between the amounts of dots and triangles is set at 2, (v) the entire task has been shortened, (vi) in the third and last block, rules switch every 3 trials (instead of every 4 trials). Blocks 1 and 2 are nonswitch and consist of 30 trials (preceded by 5 practice trials). The third block (switch) consists of 93 trials (63 non-switch and 30 switch) and is preceded by 15 practice trials. Every trial starts with a blank screen for 1000 ms, followed by an empty 4 x 4 grid presented for 1000 ms, after which the dots or triangles appear and remain visible until a response is given. Feedback is only given during practice trials (“correct,” “wrong,” and “too fast”), and appears for 500 ms directly after a response is given, before the next empty grid is presented. The ISI duration in practice and experimental trials is the same, as feedback is given during the ISI interval (500 ms). A previous study with 7- and 11-year-old children identified a relatively early maturation mechanism associated with task-set inertia and a later maturing mechanism relating to task-set reconfiguration [31]. Figure 1 shows how the testing laptops and response button boxes are best setup at the elementary school.

### Questionnaires

#### Children

In addition to EFs, we were also interested in more behavioral outcomes, for which we compiled a Web-based questionnaire (pilot-tested: 1 hour to complete) comprising:

##### Feel-KJ

This questionnaire measures 15 (mal)adaptive emotion regulation strategies in relation to anxiety, sadness, and anger: (1) Problem Solving (eg, “I try to change what makes me angry”), (2) Distraction (eg, *when I am sad…* “I do something fun”), (3) Forgetting (eg, “I try to forget what makes me angry”), (4) Acceptance (eg, “I make the best of it”), (5) Humor Enhancement (eg, I think about things that make me happy”), (6) Cognitive Problem Solving (eg, I think about how I can solve the problem”), (7) Reevaluation (eg, “I tell myself it is nothing important”), (8) Giving Up (eg, “I don’t want to do anything”), (9) Withdrawal (eg, “I don’t want to see anyone”), (10) Rumination (eg, “I cannot get it out of my head”), (11) Self-Devaluation (eg, “I blame myself”), (12) Aggressive Actions (eg, “I get into a quarrel with others”), (13) Social Support (eg, “I tell someone how I am doing”), (14) Expression (eg, “I express my sadness”), and (15) Emotional Control (eg, “I keep my feelings to myself”). There is a total of 90 items (2 items x 15 strategies x 3 emotions). In a previous research, exploratory factor analysis has suggested that the first 7 (1-7) strategies can be classified as Adaptive Emotion Regulation, the next 6 ( 8-13) as Maladaptive, and that the remaining Social Support, Expression, and Emotional Control strategies could not be classified as Adaptive or Maladaptive Emotion Regulation [32].

Internal consistency has repeatedly been found to be high, also among children [33]. We included the Dutch version [33] of the FEEL-KJ to be used in our outcome measurement.

##### Strengths and Difficulties Questionnaire (SDQ)

The SDQ is a behavioral screening questionnaire that assesses 3 domains of children’s overall mental health: social, emotional, and behavioral functioning [34]. A total of 25 items are evenly distributed among 5 scales: (1) Conduct Problems (eg, lying and stealing: “I am often accused of lying or cheating”), (2) Inattention Hyperactivity (eg, impulsivity and concentration problems: “I am easily distracted”), (3) Emotional Problems (eg, unhappy mood and worries: I am often unhappy, down-hearted or tearful), (4) Peer Problems (eg, being bullied: “Other children or young people pick on me or bully me”), and (5) Prosocial Behavior (eg, sharing and being helpful: “I am helpful when someone is hurt, upset or feeling ill”). Items are rated on a 3-point Likert Scale, with 0 representing “not true,” 1 representing “somewhat true,” and 2 representing “certainly true.” Several items are reverse-scored. The first 4 subscales summed up together give a total difficulty score ranging from 0 to 40, with a higher score indicating more difficulties. The Prosocial Behavior subscale reflects the strengths of the individual. The SDQ is an internationally approved instrument of value due to its brevity and psychometric properties [35]. In terms of internal consistency, one study among 7- to 15-year-old Finnish children and adolescents reported a Cronbach alpha of .71 [36]. We included the Dutch version of the SDQ [37] to be used in our outcome measurement.

##### Attention-Related Cognitive Errors Scale (ARCES)

The ARCES consists of 12 questions measuring everyday mistakes as a result of not paying sufficient attention to the task at hand, also called mind-wandering [38]. Example questions include: “I make mistakes because I am doing one thing and thinking about another” and “I fail to see what I am looking for even though I am looking right at it.” The 12 items are rated on a 5-point Likert Scale ranging from 1 representing “never” to 5 representing “very often.” A mean score is computed for each individual by summing the scores of all questions. The ARCES has been rigorously validated and normed [38]. We included a back-translated Dutch version of the ARCES, with some questions adapted to make them suitable for children, to be used in our outcome measurement.

##### Physical Activity Questionnaire-Child (PAQ-C)

The PAQ-C is an instrument used to assess self-reported levels of physical activity during the past 7 days for 8- to 14-years-old students [39]. The PAQ-C provides a summary physical activity score derived from 9 items, each scored on a 5-point scale. Examples include: “In the last 7 days, during your physical education (PE) classes, how often were you very active (playing hard, running, jumping, throwing)? Response options (check one only) are: “I don’t do PE,” “hardly ever,” “sometimes,” “quite often,” “always.” Or, “In the last 7 days, how many evenings did you do sports, dance, or play games in a very active way?” Response options are (check one only) “none,” “1 time last week,” “2 or 3 times last week,” “4 or 5 times last week,” “6 or 7 times last week.” The scale concludes with a small agenda (7 week days) on which the child marks how often he or she did physical activity for each day last week (“none” to “very often”). Excellent content validity, acceptable interitem reliability and a moderate to good strength of interrater agreement has been found for the Dutch PAQ-C [40].

##### Dietary Habits

The same eating behaviors as were addressed in the eHealth module of this study (consumption of vegetables, fruit, sugary beverages, and unhealthy snacks) are briefly assessed using 2 items per behavior taken from an existing questionnaire [41]. For example: “How many days in the past week did you eat fruit?” and “How many pieces of fruit on average did you eat per day?”

##### Determinants of Dietary Habits and Physical Activity

Attitude, Social Influences, and Self-Efficacy are measured for the 5 behaviors targeted in the eHealth module (physical activity, consumption of vegetables, fruit, sugary beverages, and unhealthy snacks). For example: Attitude: “Eating 3-4 serving spoons of vegetables a day would be (1 “very unpleasant” – 7 “very pleasant”) for me”; Self-Efficacy: “I am confident I would be able to eat at least 2 pieces of fruit each day, if I wanted to” 1 “very untrue” – 7 “very true” [20].

#### Demographics

The questionnaire package started with a short demographic background (age, school, country of birth child and parents, gender, dominant hand [as a control variable for the EF battery response button box]). Further demographic details were collected from the parents (Socioeconomic Status—a correlate of EF [42], medication).

To ensure maximal participation, both the EF test battery and the Web-based questionnaire were administered at school. Moreover, 30 identical laptops and response button boxes were used to ensure maximum accuracy in measuring reaction times. Administration of the task battery and questionnaires took place on different days, to minimize load and maximize children’s motivation to keep performing well. Everyone was measured within a span of 5 weeks, right before the start of the intervention and immediately after it ended. Trained research assistants (graduated psychology masters) were present at all times to supervise and instruct in the classrooms, as well as help slow readers work their way through the survey, when needed (this only happened a few times). All questionnaires had been normed for our age group, indicating readability should be decent. The task battery was administered in small groups of 5, the questionnaire in the entire group at once. While completing the computer task battery in small groups, children wore special noise-canceling headphones to avoid distraction. Furthermore, scores below chance levels (which indicates random responses) will be removed from the dataset. The strain on children was not small by any standards, but satisfactory pilot-testing and similar experiences in the field by supervising professors on the project team gave us confidence that the setup of measuring would run smoothly—which it did. The measurements concluded with a brief assessment of weight and height (body mass index, BMI). These anthropometrics were measured using standard procedures [43]. Both height (using the SECA 213 stadiometer) and weight (using the SECA 877 scales) were assessed without shoes or heavy clothing to the nearest 1 mm and 0.1 kg, respectively. BMI was calculated as weight/height squared (kg/m^2^) and Z-scores from age- and sex-specific reference values. Academic performance will also be taken into account during analysis using standardized national test scores (ie, CITO, the most widely used test in the Netherlands, designed by Centraal Instituur voor Toetsontwikkeling).

#### Teachers

Teachers are a valuable source of information regarding the children but were not included in the measurements because their time is very limited and scoring an entire class group is time-consuming. However, during the program implementation, close contact between teachers and the development team was maintained, to gain insight in the workings and feasibility of the intervention materials. Continuous feedback was encouraged and welcomed, to further improve and fine-tune the games and activities. After the intervention had taken place, an extensive (process) evaluation followed, including an assessment of fidelity and completeness.

#### Parents

We did include parents, for whom we composed a Web-based questionnaire pertaining questions about their child’s behavior. This was mainly because self-report questionnaires given to 9- to 11-year-old children could not provide an accurate overview. Note: we will only describe the BRIEF-parent scale in the following overview, as the rest of the measurements are similar to those administered directly to the children, but then from the parent *about* their child.

The Web-based questionnaire for parents included:

- Strengths and Difficulties Questionnaire
- Behavior Rating Inventory of Executive Function (BRIEF-parent). The BRIEF is an 86-item rating scale developed to assess, via parent and teacher reports, manifestations of executive function in the everyday lives of children aged 5-18 years [44]. The BRIEF yields an overall Global Executive Composite score composed of 2 indexes called the Behavioral Regulation Index (child’s ability to Inhibit [impulses], Shift [between tasks], and Emotional Control) and the Metacognition Index (child’s ability to Initiate [start a task], Working Memory, Plan/Organize, Monitor [their own behavior], and Organization of Materials)
- Attention-Related Cognitive Errors Scale (ARCES)
- Child’s physical activity and dietary habits
- Demographics (including socioeconomic status)

### Statistical Analyses

As it was not feasible to estimate population means or SDs with our new computer task battery (designed to measure our main outcome, EF’s), and we aimed to include as many schools as was practically possible, a power analysis for this study is not included. Unfortunately, we were not able to reach the recommended critical sample size of 50 at the group level [45]. Statistical techniques to compensate for this potential lack of power for certain multilevel analyses will be considered and investigated carefully. We will assess the effectiveness of the TyM program using multilevel analyses (3 levels: student [n=800], class [n=34], and school [n=13]), to adjust for clustering of observations within a class or school. Randomization took place at the school level. The potential mediation our primary outcome, EF, exerts on our secondary outcomes (healthy behaviors: physical activity and healthy eating) will be investigated using the Baron and Kenny’s method [46], adding EFs as a covariate. Note that the intervention and measurements have been concluded, but the data have not yet been completely analyzed.

## Results

Results are expected to be submitted for publication in 2018.

## Discussion

This study design protocol describes the cluster randomized trial to test the effectiveness of TyM, an intervention designed to enhance EFs among elementary school children aged between 9 and 11 years. Multiple modules (focused physical activity, cognitive games, and socioemotional development) are combined in TyM to increase the chances of EFs to improve and broad transfer to occur (translation of cognitive effects into daily life improvements, such as physical activity, adaptive emotion regulation, and healthy eating). Although broad transfer has not yet been demonstrated in the field, leading experts suspect combining approaches may yield promising results [2,10-12]. TyM is novel in the sense that it combines a physical-, cognitive-, and socioemotional approach in one integrated program. The development of the intervention and why EFs are of such cardinal importance to a healthy and happy life are described in a separate paper (J Bervoets et al, unpublished data, 2018). EFs are the very core of the entire project, which is why careful attention was paid to the measurement of these. An extensive 1 hour computer task battery was designed, with 2 tasks per sub-EF; impulse control: Flanker and Go/No-Go; working memory: N-Back and Running Span; cognitive flexibility: Attention Switching Task and Dots/Triangles. Additionally, questionnaires measured attention, concentration, emotion-regulation, social/behavioral problems, physical activity, and healthy eating. BMI was also measured. The TyM data will be enriched with academic performance and socioeconomic status from a provincial database to investigate the relations between our variables of interest. We hope to shed more light on the intricate connections between EFs, healthy behaviors, academic performance, and emotion regulation. Considering the vast array of possible positive outcomes of training EFs, efforts in this direction are warranted and justified. Given the intense nature of optimal cognitive training, and significant personal differences, combining various approaches in a playful context seemed like something teachers and pupils could continue doing wholeheartedly for a longer period of time.

## Abbreviations

ARCES: Attention-Related Cognitive Errors Scale
AST: Attention Switching Task
BMI: body mass index
BRIEF: Behavior Rating Inventory of Executive Function
CITO: Centraal Instituur voor Toetsontwikkeling (Central Institute for the Development of Tests)
EF: executive function
FEEL-KJ: Fragebogen zur Ehrebung der Emotionsregulation bei Kindern und Jugendlichen (Questionnaire for the Assessment of Emotion Regulation among Children and Adolescents)
ISI: interstimulus interval
PAQ-C: Physical Activity Questionnaire for Children
SDQ: Strengths and Difficulties Questionnaire
SRI: stimulus and response incongruent
TyM: Train your Mind
